# Prospectively Collected Characteristics of Adult Patients, Their Consultations and Outcomes as They Report Breathlessness When Presenting to General Practice in Australia

**DOI:** 10.1371/journal.pone.0074814

**Published:** 2013-09-17

**Authors:** David C. Currow, Katherine Clark, Geoffrey K. Mitchell, Miriam J. Johnson, Amy P. Abernethy

**Affiliations:** 1 Discipline, Palliative and Supportive Services, Flinders University, Bedford Park, South Australia. Australia; 2 University of Newcastle, Callahan, Newcastle, New South Wales, Australia; 3 Hunter New England Area Palliative Care Service, Calvary Mater Hospital, Waratah, New South Wales, Australia; 4 School of Medicine, University of Queensland, Ipswich Clinical School, Ipswich, Queensland, Australia; 5 Hull York Medical School, the University of Hull, Hull, United Kingdom; 6 Duke University Medical Center, Durham, North Carolina, United States of America; University of Liverpool, United Kingdom

## Abstract

**Introduction:**

Breathlessness is a subjective sensation, so understanding its impacts requires patients’ reports, including prospective patient-defined breathlessness as a reason for presenting to general practitioners (GP).The aim of this study was to define the prevalence of breathlessness as a reason for GP consultations while defining the clinico-demographic factors of these patients and the characteristics and outcomes of those consultations.

**Methods:**

Using nine years of the Family Medicine Research Centre database of 100 consecutive encounters from 1,000 practices annually, the patient-defined *reason* *for* *encounter* ‘breathlessness’ was explored using prospectively collected data in people ≥18 years with clinical data coded using the International Classification for Primary Care V2. Dichotomous variables were analysed using chi square and 95% confidence intervals calculated using Kish’s formula for a single stage clustered design.

**Results:**

Of all the 755,729 consultations collected over a nine year period from 1 April, 2000, 7255 included breathlessness as a *reason* *for* *encounter* (0.96%; 95% CI 0.93 to 0.99) most frequently attributed to chronic obstructive pulmonary disease. Only 48.3% of GPs saw someone reporting breathlessness. The proportion of consultations with breathlessness increased with age. Breathlessness trebled the likelihood that the consultation occurred in the community rather than the consulting room (p<0.0001) and increased 2.5 fold the likelihood of urgent referral to hospital (p<0.0001). Of those with breathlessness, 12% had undiagnosed breathlessness at the end of the consultation (873/7255) with higher likelihood of being younger females.

**Discussion:**

Breathlessness is a prevalent symptom in general practitioner. Such prevalence enables future research focused on understanding the temporal pattern of breathlessness and the longitudinal care offered to, and outcomes for these patients, including those who leave the consultation without a diagnosis.

## Introduction

By definition, breathlessness is subjective [1]. Any evaluation of breathlessness must therefore be defined by patients themselves. A vehicle for studying patient-defined presentations to primary care is the national Bettering the Evaluation and Care of Health (BEACH) data set of the Family Medicine Research Program at the University of Sydney because it systematically captures a representative sample of the *reason(s*)* for encounter* as identified by patients as they present to primary care, in contrast to most health service studies that focus on the diagnosis made by clinicians at the conclusion of consultations[2].

Generally, health services are well geared to respond to acute breathlessness (e.g. pneumonia, acute worsening of cardiac function) and acute-on-chronic (e.g. an acute exacerbation of chronic obstructive pulmonary disease) presentations.

There are also a large number of people who have chronic breathlessness at rest or on minimal exertion despite optimal treatment of the underlying causes, now termed chronic refractory breathlessness [3]. As an evidence base emerges for the diagnosis and symptomatic treatment of chronic refractory breathlessness, it is necessary to understand how these people interact with health services and, in subsequent work, to understand the clinical outcomes from these encounters. In the Australian health system, this requires an understanding of presentations to primary care as all care is centred around or brokered by general practitioners with the exception of use of the Emergency Department.

The aim of this study is to describe the interactions that occur in primary care that relate to patient-defined breathlessness including the prevalence, patient characteristics, consultation characteristics, clinical evaluation, and outcomes of consultations across the community. The study is therefore a first step in understanding the interactions of people presenting with breathlessness and primary care, to inform more detailed research especially of chronic refractory breathlessness [4]. Hypotheses included that there:

- are characteristics that distinguish people with breathlessness from other people presenting to primary care;
- are differences in presentations, consultations and outcomes for people with breathlessness when seen in consulting rooms compared to home visits; and
- is an identifiable sub-group of people with breathlessness without a diagnosis at the end of the consultations.

## Methods

### Ethics Statement

Ethical approval for the BEACH program during 2000-2009 was provided by the Human Ethics Committee of the University of Sydney and the Ethics Committee of the Australian Institute of Health and Welfare. Individual written informed consent was not required by the ethics committees because these data were collected for the purpose of subsequently analysing the characteristics of the aggregated consultations and their outcomes. Patient data were supplied by general practitioners only after each patient was provided with an information sheet and gave verbal consent for the data relating to their consultation to be included. No individually identifying patient characteristics are collected nor reported.

### Setting

General practice in Australia is the first contact with the health system, and the gatekeeper to specialist and allied health service with the exception of emergency departments. On average, each Australian visits a GP between 4 and 5 times annually. GPs visits make up 80% of all doctor encounters, with the remaining 20% being specialist encounters [5].

The annual survey of 1000 general practices (randomly selected from the Federal Government Department of Health and Ageing’s register) each entering 100 consecutive patients’ consultations using standardised data forms generates a database of about 100,000 patient encounters per year. The data fields have remain unchanged over the course of the program and are not at the discretion of the current researchers. The data provide a snapshot of the nature of GP consultations, specifically the number and type of clinical problems presenting, and diagnostic, treatment and referral decisions that GPs subsequently make. This 0.1% of the total nationally funded Medicare (national universal health care costs reimbursement scheme) claims for GP services is directly representative of general practitioners’ clinical workloads. To be approached, GPs had to be ‘active’ which was defined as having claimed at least 375 items through Medicare in the preceding three months.

### Population

All clinical encounters with people aged 18 years or older are included, given that childhood breathlessness is likely to be from a different range of aetiologies and is therefore beyond the scope of the current analysis.

### Data

Data routinely collected at point of care included: age and gender of patient; up to three (patient-defined) reason(s) for presenting to the GP (reason for encounter (RFE)) [6]; the diagnoses or problems managed (up to 4) by the completion of the consultation; new or existing clinical problem for that patient; the Medicare item numbers claimed at the consultation (which identifies the length of the consultation and the place where the consultation took place); and the outcomes (including the ordering of imaging and/or pathology and referral to other practitioners).*Reason* *for* *encounter*, problems managed, imaging and pathology investigations ordered, and referrals made were classified according to the International Classification of Primary Care version 2 [7]. *Reasons* *for* *encounter* included in the coding of breathlessness included *shortness* *of* *breath/dyspnoea* (ICPC-2 code RO_2_) and *breathing* *problems, other* (code RO4).

### Analysis

Descriptive data are presented. The calculations of 95% confidence intervals incorporate the study design (single stage, clustered study design) according to Kish’s formula [8]. Categorical variables were analysed using p values based on F statistics, corrected for design effect. Significance was accepted for p values less than 0.05 and 95% confidence intervals are cited. The statistical software package SAS 9.2 was used in the descriptive analyses (SAS Institute. SAS/STAT® User’s guide, Version 9. Cary, NC: SAS Institute Inc, 2002-2003.), and Stata version 11.0 in statistical analyses (StataCorp. 2009. Stata Statistical Software: Release 11. College Station, TX: StataCorp LP).

The paper complies with STROBE consensus guidelines for reporting an observational study[9].

## Results

Responses in this study are included from 8,847 participating GPs over nine years (1 April, 2000 to 31 March, 2009), who provided cross-sectional data for 885,400 patient encounters. Of these, 129,671 were in people younger than 18 years of age, and these data were excluded from the analyses.

Practitioner characteristics (n=8847)

The majority of GPs were male (70.1%) and the majority had been in practice for 20 years or more (57.5%). Most (73.5%) worked 6-10 sessions (half days) per week in group practices (75.0%) of 2-9 practitioners with 4.6% of respondents (149) conducting more than 50% of their consultations in languages other than English. Only 4,273 (48.3%) of the participating GPs recorded seeing someone who presented with breathlessness during their recording periods.

### Prevalence of breathlessness in Australian general practice consultations

Of these 755,729 encounters, patients presented with breathlessness as at least one *reason for encounter* in 7,255 consultations (0.96%; 95% confidence interval (CI) 0.93-0.99). (Table 1)

**Table 1:** Patient characteristics by of people presenting with breathlessness as one of the *reasons for encounter* to general practice in Australia 2000-2009 compared to all other consultations.

Patient characteristics n (Col %)		All adult consultations n= 753, 662*		p value
		Breathlessness is one reason for encountern=7,255	All other consultations n=746,407	
Patient characteristics				
**Prior status with this practice**	New patient	501 (7.0)	60,372 (8.2)	0.002
	Seen before	6,615 (93.0)	673,167	
			(91.8)	
	Missing	139	12,868	
**Sex**	Male	3,290 (45.8)	292,078	<0.001
			(39.4)	
	Female	3,897 (54.2)	448,709	
			(60.6)	
	Missing	68	5,620	
**Age**	18-64 years	3,070 (42.3)	513,574	<0.001
			(68.8)	
	≥65 years	4,185 (57.7)	232,833	
			(31.2)	

Missing data removed from analysis. * additionally, there were 129,671 people under the age of 18 whose data were not analysed.

### Patient characteristics of people who were breathless (n=7255)

Of the people who had a *reason for encounter* of breathlessness (n=7255), 45.8% were male (95% CI 44.6 to 47.0). Breathlessness as a *reason for encounter* increased with age. The most prevalent age group was 75 years or older, accounting for 36.8% of consultations where breathlessness was a *reason for encounter* (Table 2).

**Table 2:** Age and gender distribution of people presenting with breathlessness as one of the *reasons for encounter* to general practice in Australia 2000-2009.

Gender of patient n row % (95% CI)	Patient age group					Total n=7,255 n col% (95% CI)
	18-24 years	25-44 years	45-64 years	65-74 years	75+ years	
Male	111 **3.4** (2.8-4.0)	416 **12.6** (11.5-13.8)	824 **25.1** (23.5-26.6)	781 **23.7** (22.2-25.3)	1,158 **35.2** (33.4-37.0)	3,290 **45.8** (44.6-47.0)
Female	163 **4.2** (3.5-4.8)	591 **15.2** (14.0-16.4)	936 **24.0** (22.6-25.4)	721 **18.5** (17.2-19.8)	1,486 **38.1** (36.4-39.8)	3,897 **54.2** (53.0-55.4)
Total	274 **3.8** (3.3-4.3)	1,007 **14.0** (13.1-14.9)	1,760 **24.5** (23.4-25.6)	1,502 **20.9** (19.9-21.9)	2,644 **36.8** (35.5-38.1)	7,187

Missing data removed from analysis (n=68).

### Patients’ reasons for encounters

Of the 7255 encounters, 33.3% included breathlessness as the only *reason for encounter* recorded, while breathlessness was accompanied by one (38.7%) or two (28.0%) additional *reasons for encounters* for the remainder. Where there were identified problems in addition to breathlessness (n=4826), these included additional *reasons for encounter*: cough (55.0%); chest pain (17.6%); weakness/tiredness (13.5%); depression (3.9%); and anxiety (3.8%).

### Consultation characteristics

Of the clinical encounters where breathlessness was a reason for encounter, 62.4% were standard length consultations (<20 minutes with limited physical examination), while 21.8% were long consultations (20-40 minutes) and 1.9% were greater than 40 minutes (Table 3).

**Table 3:** Consultation characteristics by breathlessness status at the encounter of people presenting with breathlessness as one of the *reasons for encounter* to general practice in Australia 2000-2009.

Consultation characteristics n (Col %)		All adult consultationsn= 753, 662		p value
		Breathlessness is one reason for encounter n=7,255	All other consultations n=746,407	
**At least one imaging ordered at encounter**	Yes	1,602 (22.1)	61,176 (8.2)	<0.001
	No	5,653 (77.9)	685,231 (91.8)	
**At least one pathology ordered at encounter**	Yes	1,520 (21.0)	140,365 (18.8)	<0.001
	No	5,735 (79.0)	606,042 (81.2)	
**Type of Consultation**	Level A or B MBS/DVA items^d^	4,633 (69.8)	529,851 (77.1)	<0.001
	Level C or D MBS/DVA items^d^	1,722 (25.9)	90,558 (13.2)	
	Other ^a^	283 (4.3)	66,510 (9.7)	
**Place of consultation**	Surgery	5,724 (86.2)	597,723 (87.0)	<0.001
	Home or RACF	512 (7.7)	18,722 (2.7)	
	Not definable^c^	402 (6.1)	70,474 (10.3)	
	Missing ^b^	617 (8.5)	59,488 (8.0)	

Missing data removed from analysis.

RACF − residential aged care facility; MBS − Medicare Benefits Schedule; DVA − Department of Veterans’ Affairs.

(a) MBS/DVA items other than level A, B, C, D items, and workers compensation claim, or other paid (hospital, state etc), and no charge.

(b) No MBS/DVA items or type of consultation (workers compensation claim, other paid, or no charge) recorded.

(c) MBS/DVA items and other types of encounter (workers compensation claim, or other paid [hospital, state etc], or no charge) that do not designate the place of consultation.

(d) Level A <5 minutes (uncomplicated consultation e.g. immunisation); Level B <20 minutes with limited physical examination; Level C long consultations (20-40 minutes); and Level D >40 minutes.

In community consultations (home 62%, residential aged care facility 38%), breathlessness was three times as likely to be the *reason for encounter* (512/19,235; 2.7%; p<0.001) as the rate in clinic-based consultations. Patients presenting with breathlessness were less likely to be new to the practice than patients at all other consultations ((7.0%, (95%CI 6.3 to 7.8) compared with 8.2%; 95% CI 8.0 to 8.5; p<0.001).

### Consultation outcomes - problems managed

The most frequent diagnoses associated with patient-defined presentations of breathlessness were chronic obstructive pulmonary disease (10.4%), asthma (9.6%), heart failure (9.4%), hypertension (4.1%), acute bronchitis / bronchiolitis (4.0%), ischaemic heart disease (3.0%), sleep disturbance (2.4%) and anxiety (2.1%).

### Consultation outcomes – investigations and prescribing

A total of 1,905/7,255 (26.3%) radiological tests were ordered and 4,526 pathology tests (62.4%) where breathlessness was a *reason for encounter* (Table 4). No significant differences were seen in imaging rates by gender or age.

**Table 4:** Consultation outcome by place of consultation of people presenting with breathlessness as one of the *reasons for encounter* to general practice in Australia 2000-2009.

Outcome of consultation n (Col %)		Place of consultation			Total encounters at which breathlessness was a RFE n=7,255	p value
		Surgery	Home or RACF	Other than listed*		
**Referral to hospital**	Yes	139 (2.4)	31 (6.1)	50 (12.4)	220	<0.001
	No	5,585 (97.6)	481 (93.9)	352 (87.6)	6,418	
**Total**		5,724	512	402	6,638	

Missing data removed from analysis (n= 617; 8.5%); RACF-residential aged care facility.

^*^Includes telephone consultations, indirect encounters and seeing people in other hospitals.

### Consultation outcomes – prescribing

On average, 3 medications were prescribed for each 2 consultations where breathlessness was one of the *reasons for encounter*.

### Consultation outcome – referral

A total of 1,675 people were referred to other services of whom 359 were referred to an Emergency Department (4.9%; Table 4). When analysed by place of consultation, if seen in a doctor’s surgery, 2.4% (95% CI 2.0 to 2.8) were referred to hospital acutely while this rose to 6.1% (95% CI 3.8 to 8.3%; p<0.0001) for community-based consultations. Referral to specialists occurred in 1,139 cases (15.7%) and to allied health on 177 occasions (2.4%).

### Reason for encounter was breathlessness but no diagnosis was recorded (n=873)

Twelve percent of people with breathlessness as a *reason for encounter* had no recorded diagnosis for breathlessness at the end of the consultation (873/7255). These people were likely to be younger (18-64; 15.1% ; 95% CI 13.8 to 16.4 compared with 9.8% (95% CI 8.8 to 10.7 for people ≥65) and female (13.2% ; 95% CI 12.1 to 14.3; males 10.7%; 95% CI 9.6 to 11.8%; p = 0.001). There was no difference whether this was a first presentation to this practitioner or not (12.2% versus 12.0%). Patients with breathlessness were more likely to have radiological imaging ordered if this was a new problem (47.7% (95% CI 42.9 to 52.5) than an existing problem (34.1% (95% CI 29.6 to 38.6; p<0.0001)). Likewise, ordering pathology was more likely if this were a new problem (44.2%; 95% CI 40.8 to 47.5) than an existing problem (19.1%; 95% CI 18.0 to 20.2; p<0.0001).

## Discussion

The study found that breathlessness was encountered in one in one hundred consultations in adults with two distinct groups arising: a younger cohort where a diagnosis was not made and an older cohort who were likely to have causes that were actively investigated and treated. Active investigation and management were more likely to occur in new presentations. Of concern is that only 2.4% of people identifying breathlessness as at least one reason for attending their general practitioners were referred to allied health practitioners[10]. Patients requiring a community visit were more likely to have breathlessness as the *reason for encounter*, and people with breathlessness in these settings were more likely to be referred to the Emergency Department. More than half of the general practitioners did not see anyone with breathlessness during their data collection periods.

Approximately 90% of Australians will visit a doctor in any given year [11]. Despite no compulsory registration with individual practices nor practitioners, 92% would always use the same practice and more than two-thirds the same practitioner as their preference [11] allowing for continuity of care when investigating and treating symptoms such as breathlessness.

This study confirms other work in breathlessness. Attributable causes are predominantly due to lung disease, followed by cardiac disease [12,13,14]. Given under-diagnosis and under-treatment of breathlessness and its causes [13,14,15], actively seeking a diagnosis for the underlying cause(s) using standardised algorithms [13,14] improves outcomes. Despite such an approach, for many people, breathlessness will persist. In a study of 123 consecutive consultations in primary care for *chronic* breathlessness (defined as breathlessness persisting for >8 weeks), a cause was found in 99%, treatment of which improved the breathlessness in only 63% leaving 37% of people with ongoing symptoms that were, in essence, ‘refractory’. [16]

A key strength of this study was the use of patient-reported problems as the basis for defining the cohort, as breathlessness is ultimately a subjective symptom. In contrast to almost all other studies of service delivery in primary care which focus on the diagnosis at the end of the consultation, this study focuses on symptoms that motivate patient to initiate their GP consultations.

One other study has considered the *reason for encounter* of dyspnoea in primary care although they covered all ages, not just adults, and it was a secondary analysis of data from two studies [17]. The cohort covered by Frese et al. also had a prevalence of dyspnoea of around 1% of all consultations with almost 50% of cases being for a new onset of dyspnoea, but with much lower rates of referral to hospital. Unlike the study by Frese et al., the Australian cohort is an unselected primary care population, whereas Frese et al. selected populations using specific selection criteria.

In this report of Australian data, women were more likely to report breathlessness. Gender differences reflect a small but consistent difference seen in other studies [3,18,19], although this difference is no longer apparent if the analysis controls for mood[20].

Referral rates for hospital assessment differ by place of consultation. Attending the doctor’s surgery in person reduced the likelihood of hospital referral in contrast to home visits. Being able to get to the surgery in itself, self-selects a level of wellbeing that is less likely to be seen in people requiring a community visit. Breathlessness is in the top ten reasons for adults presenting to the emergency department [21].

There was a group of people who had breathlessness without a diagnosis at the end of the consultation. Despite no diagnosis related to breathlessness, less than one half of these people had investigations (radiology or pathology) as an outcome of the consultation. This may relate to: the breathlessness not being perceived as serious by the patient, the doctor or both; that the serious causes of breathlessness had been excluded on clinical grounds; the judgement of the practitioner may be that a formal diagnosis is not possible on the clinical evidence and further time will lead to clarification of the problem, or resolution if the cause is thought to be self-limiting; or that breathlessness was not the primary reason for the consultation and other issues therefore took precedence. The rate found is similar to Pedersen et al. who demonstrated that up to 27% of people with breathlessness persisting for more than 8 weeks may not have an obvious cause for their dyspnoea [13].

One key reason to better understand the prevalence of persisting breathlessness as a symptom is the effectiveness of treatments introduced in recent years to control reversible causes of breathlessness. As lung disease is the most prevalent cause of breathlessness across the community [12], reducing community rates of smoking, and use of inhaled long acting beta agonists, long acting inhaled steroids and anticholinergic agents have been remarkably successful in reducing the morbidity in this setting. Likewise, effective treatment of early heart failure with routine use of beta blockers, angiotensin converting enzyme (ACE) inhibitors, anti-platelet medications and lipid lowering agents after a myocardial infarction has dramatically reduced the incidence of further myocardial damage and subsequent breathlessness as a cause of chronic cardiac-related breathlessness. Routine immunisation against influenza, pneumococcus and *haemophilus influenzae*, and systematic changes in general practice to develop management plans for people with known chronic lung and heart conditions may also have contributed to this rate.

### Limitations - data

These data do not distinguish between acute, chronic and acute-on-chronic breathlessness or allow any inference about the severity of breathlessness. Given the structure of the database, no distinctions can be made about whether appointments were urgent or long-planned. Further, given that this is only a cross sectional interrogation of a primary care database, the subsequent clinical care of these people (no follow-up, GP follow-up, referral to the Emergency Department, referral for specialist input, referral to allied health practitioners) requires linkage to their other health service databases with identifiable data, something which is explicitly beyond the scope of the available data.

Chronicity may be under-reported as it may be more likely that new symptoms are reported as the *reason for encounter* with less emphasis on chronic problems as people adapt to the limitations of exertional breathlessness. Patients may no longer emphasise a symptom to which they have accommodated through adjusting their activities of daily living [22]. This would be consistent with the strikingly concordant accounts of 18 people with COPD interviewed regarding the health-seeking trajectory for their chronic breathlessness [23]. All participants independently gave the sequence of events as one of delay in seeking medical help for breathlessness until crisis point. After a diagnosis was given, management of the breathlessness was inadequate, despite treatment of the COPD, and participants dealt with the daily burden of increasing breathlessness largely without recourse to medical help

### Limitations – sample

This is a well-established data sampling system with set fields collected over the entire life of the program. Participation rates remain high despite structural changes in general practice occurring across the study period such as the increasing numbers of general practitioners working part time. The threshold for participation is low enough that the broadest spectrum of practice types is included.

### Further research

Most importantly, this study gives a useful estimate of prevalence of breathlessness in primary care which can inform the design of studies to investigate the temporal patterns of presentations due to breathlessness (acute, acute-on-chronic and chronic (refractory)), its severity (especially in limitations to the activities of daily living) and define the current approaches to each of these differing clinical entities. Given a prevalence of 1%, such studies are feasible if thoughtfully designed. Future studies will be able to track subsequent investigations, health service utilisation and clinical outcomes prospectively in order to understand the longitudinal outcomes for this patient cohort.

It will be important in prospective work with emergency department data to find out what happens to people referred directly by their general practitioners, and whether this population contrasts with those who self-refer directly to the emergency department or who are taken there directly by ambulance, both with and without consultation with their general practitioner (even if this is only a patient making a phone call to the GP’s surgery). How many of them are admitted, and how many of them are discharged directly from the emergency department? Of those sent home, were investigations done that are not readily accessible in the community[13]? Studies so far examining the role of breathlessness in presentation to the emergency department, indicate that breathlessness greatly increases the chance of admission to hospital [24,25]. Additional prospective data may also be of use for the group where a cause of breathlessness was not found, and investigations nor referrals to other clinicians were instigated.

### Implications for practice

A sub-group of people in this study will have refractory breathlessness defined as persistent breathlessness at rest or on minimal exertion when all reversible factors have been treated. Given that 8.9% of the population has chronic breathlessness of this magnitude [3], how many people with refractory breathlessness are having this diagnosis made actively and being treated with evidence-based interventions that have been shown to reduce the sensation of breathlessness without otherwise compromising their health[26,27,28]? Understanding how to identify this sub-set of people and ensure that the chronic symptom of breathlessness at rest or on minimal exertion is being treated is a key challenge in reducing the impact of this symptom across the community.

## Conclusions

These data showed that breathlessness was reported by patients in 1% of primary care consultations. This is the first study to examine prospectively breathlessness as a patient-defined *reason for encounter* in a large national database of contemporaneously documented primary care consultations. It is an important first step which demonstrates that this method of interrogating “real-life” clinical encounters was successful in achieving the study objectives. This can now provide the basis for further prospective investigations of the subsequent course of people who present to primary care with breathlessness.
